# Fibular hydatid cyst

**DOI:** 10.4103/0019-5413.33692

**Published:** 2007

**Authors:** Hamidreza Arti, Hossein Yousofi Darani

**Affiliations:** Department of Orthopedic Surgery, Kashani Hospital, Faculty of Medicine, Shahrekord University of Medical sciences, Shahrekord, Iran; * Department of Parasitology, Kashani Hospital, Faculty of Medicine, Shahrekord University of Medical sciences, Shahrekord, Iran

**Keywords:** Bone cyst, hydatid disease, pathologic fracture

## Abstract

Hydatid disease is caused by the tapeworm Echinococcus. Genus Echinococcus has different species including *Echinococcus vogeli, Echinococcus granulosus* and *Echinococcus multilucularis. Echinococcus granulosus* is the most common cause of hydatid disease in humans. This disease occurs either through direct ingestion of parasite eggs from contact with infected dogs or indirectly from the ingestion of contaminated water or food.

Infestation of hydatid disease in humans most commonly occurs in the liver (55-70%), followed by the lungs (18-35%). Bone hydatidosis however is very rare (3%). We present herein a case of hydatid cyst of the fibula, which is an uncommon site for the occurrence of this disease.

Hydatid disease is endemic in the Middle East, including Iran.^1^ Though skeletal involvement is usually secondary to hepatic or pulmonary hydatidosis, it may, on occasion, occur as the primary disease.^2^ Cases have been reported in the vertebrae, the femur, the tibia and the pelvis but have not been reported in fibular bone.^3^–^5^ Intraosseous lesions usually begin at the epiphysis and may be polycystic or may occur, though less often, in the form of a solitary hydatid cyst.^6^ The polycystic type occurs because the cyst is unable to expand and fragments causing diffuse spreading of the daughter cyst and scolices along the bone canals owing to bone rigidity. In both types of hydatid cyst, pressure absorption of the bone, with resultant thinning and fracturing and extension through the periosteum and soft tissues are known to occur.^7^ Hydatid disease of the bone is often asymptomatic for a long duration and is usually detected after a sudden fracture, secondary infection or neurovascular lesion caused by compression.^3^,^4^,^6^–^8^ A definite preoperative diagnosis without histological examination is often difficult, as there are not pathogonomonic signs. Radiographic findings, perhaps misdiagnosed as those of other lesions and immunologic test, are of limited value.^9^ We herein present a case of hydatid cyst of fibula, which is an uncommon site for occurance of this desease.

## CASE REPORT

**Figure 1:**
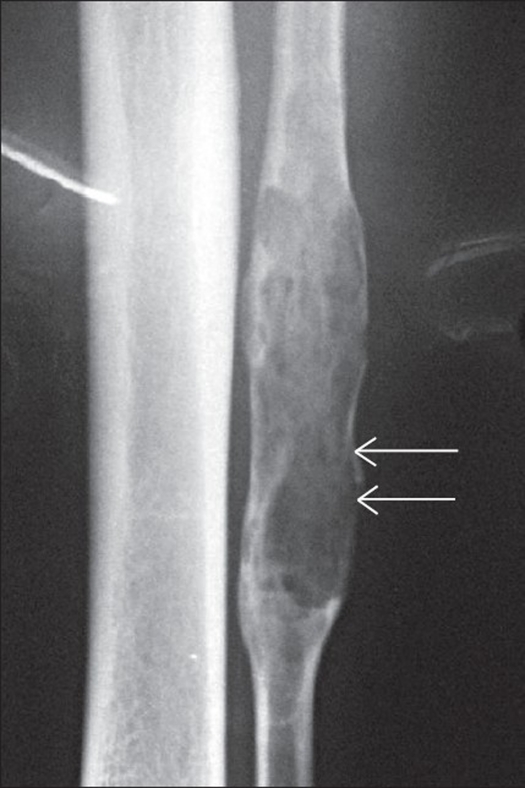
Plain radiograph (Antero Posterior view) of left fibula in a 42-year-old man showing a large cystic lesion with cortical thinning and no soft tissue extension

A 42-year-old shepherd presented with a two-week history of sudden pain and very mild swelling in the left lower leg. A tender swelling was noted at the upper to middle third of the left lateral aspect of leg. Radiographic findings revealed a multiple osteolytic lesion of the fibula with no reactive sclerosis [Figure 1]. A clinical diagnosis of a benign bone cyst was considered and an excisional biopsy was performed.

A direct lateral approach to the fibula after protection of the proneal nerve was used and surgical exposure was easily achieved. The osteotomy and resection of the bone cyst was done about 10 cm above and below its borders. After the resection of the bone cyst, the wall was opened by a curette and translucent cyst was seen.

**Figure 2:**
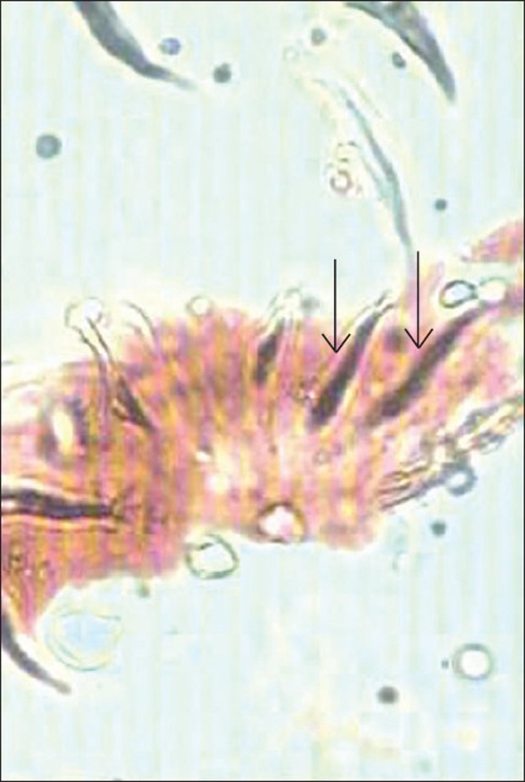
Photomicrograph (100×) of bone tissue showing scolex

Histopathology revealed the characteristic trilamellar hydatid cyst wall and scolices of *Echinococcus granulosus* scattered amidst fragments of bone and bone marrow [Figure 2] were seen.

An ultrasound of the abdomen was later performed and did not reveal hydatid cyst in the lung or the liver. The patient was treated with albendazole for three cycles at a dose of 400mg twice a day for four weeks followed by a two- week rest without therapy.^10^,^11^ The patient was followed for one year with no recurrence of the lesion.

## DISCUSSION

The disease in the bone and joint begins once the blood-borne scolex settles there. It is a very slow process and therefore, bone cysts are seldom discovered in childhood, even though infestation probably occurs at this time.^12^ There is fragmentation and conglomeration of the daughter cyst. The bone offers mechanical resistance. Due to pressure, the trabecullae are absorbed and if the cortex is breached, the cyst expands uniformly in the soft tissues. Articular cartilage and intervertebral disc offer the least resistance to growth.^13^,^14^

Disease should be suspected in cystic lesions affecting the bone, especially in endemic areas, as it may easily be misdiagnosed. It can mimic tuberculosis, simple bone cyst, sub-acute arthritis, giant cell tumors, malignant fibrous histiocytoma, myeloma and chondrosarcoma.^15^ Diagnosis of bone hydatidosis is based on roentgenographic findings and sometimes is established after surgery. Because of the poor results with medical treatment, osseous hydatidosis must be treated by a radical operation with wide excision.^16^

The purpose of this article is to alert orthopedic surgeons of this rare condition to emphasize the fact that this disease should be suspected in cystic lesions affecting any organ in the body, especially in endemic areas of the world and open and percutaneous needle biopsies should be avoided in such cases.
